# Automated quantification of interstitial lung abnormalities and emphysema on computed tomography: a predictive marker for postoperative pulmonary complications after esophagectomy

**DOI:** 10.1007/s10388-026-01208-0

**Published:** 2026-05-13

**Authors:** Seong Yong Park, Yunjoo Im, Jonghoon Kim, You Jin Oh, Joonghyun Ahn, Yeong Jeong Jeon, Junghee Lee, Jong Ho Cho, Hong Kwan Kim, Yong Soo Choi, Jae Il Zo, Young Mog Shim, Hye Yun Park, Ho Yun Lee

**Affiliations:** 1 Department of Thoracic and Cardiovascular Surgery, Samsung Medical Center, Sungkyunkwan University School of Medicine, Seoul, Republic of Korea; 2 Division of Pulmonology and Allergy, Department of Internal Medicine, Kyung Hee University Medical Center, Seoul, Republic of Korea; 3 Department of Radiology and Center for Imaging Science, Samsung Medical Center, Sungkyunkwan University School of Medicine, Seoul, Republic of Korea; 4 Department of Health Sciences and Technology, Samsung Advanced Institute for Health Sciences & Technology (SAIHST), Sungkyunkwan University, 81 Irwon-ro, Gangnam-gu, Seoul, 06351 Republic of Korea; 5 Biomedical Statistics Center, Data Science Institute, Samsung Medical Center, Samsung Medical Center, Seoul, Republic of Korea; 6 Division of Pulmonary and Critical Care Medicine, Department of Medicine, Samsung Medical Center, Sungkyunkwan University School of Medicine, Seoul, South Korea

**Keywords:** Esophageal neoplasms, Esophagectomy, Pulmonary complicatio

## Abstract

**Background:**

Postoperative pulmonary complications (PPCs), including pneumonia, acute lung injury, and acute respiratory distress syndrome, are common morbidities associated with mortality following esophagectomy. This study aimed to assess the association of chest computed tomography (CT) texture features with PPCs following esophagectomy.

**Methods:**

Between 2016 and 2022, data from 765 patients who underwent upfront esophagectomy were analyzed. Deep learning–based automatic quantifi cation was used to identify interstitial lung abnormalities (ILAs) and emphysema on the preoperative chest CT. Logistic regression analyses were performed to identify risk factors for PPC.

**Results:**

The mean age of the patients was 64.72 ± 8.27 years, and 698 (91.2%) patients were male. PPCs developed in 129 (16.2%) patients. Patients with PPCs were more likely to have current smoking status, lower lung function, and open esophagectomies than patients without PPCs. The PPC group also exhibited more emphysema (0.236% vs. 0.123%, p= 0.005) and ILAs (0.342% vs. 0.149%, p 0.001) on chest CT scans compared with patients without PPCs. Multivariable logistic analysis demonstrated that emphysema (odds ratio [OR] 1.158, p = 0.004) and ILA (OR 1.364, p 0.001) were risk factors for PPC after adjusting for other confounding factors.

**Conclusions:**

The extent of emphysema and ILA, quantifi ed by automated software, was signifi cantly associated with PPC following esophagectomy. Future research should focus on perioperative management strategies for patients with emphysema or ILA and esophageal cancer.

**Supplementary Information:**

The online version contains supplementary material available at 10.1007/s10388-026-01208-0.

## Introduction

Esophagectomy, a key component of the multidisciplinary treatment for resectable esophageal cancer, is associated with higher morbidity and mortality compared to other gastrointestinal surgeries [1–3]. Postoperative pulmonary complications (PPCs), including pneumonia, acute lung injury (ALI), and acute respiratory distress syndrome (ARDS), are the most common and severe morbidities following esophagectomy [4]. PPCs, which significantly contribute to hospital mortality [5], are independent risk factors for poor long-term survival [6–8]. Despite advanced surgical techniques, such as minimally invasive esophagectomy, and improved perioperative care, PPCs develop in 16%–23% of patients undergoing esophagectomies [2]. Thus, identifying and mitigating the risk factors for PPCs is crucial in improving surgical outcomes after esophagectomies. Known risk factors for PPCs include compromised lung function, such as reduced forced expiratory volume in one second (FEV_1_) and diffusing capacity for carbon monoxide (DL_CO_), smoking history, poor oral hygiene, suboptimal perioperative nutritional status, open esophagectomy, and perioperative steroid administration [4, 9]. Although patients with esophageal cancer routinely undergo computed tomography (CT), the association of lung abnormalities identified by CT with PPCs is unclear.

Interstitial lung abnormalities (ILAs), which are defined as incidental CT findings of lung parenchymal abnormalities, may be indicators of early interstitial lung disease (ILD) [10]. The prevalence of ILAs is 4%–9% in smokers and 2%–7% in the general population [11, 12]. ILAs are associated with rapidly declining lung function [11, 12], increased mortality [13], and higher risks of lung cancer development and poor prognosis [14]. Emphysema is another incidental CT finding. Emphysema, which is defined as an abnormal permanent enlargement of the air space, indicates the destruction of the lung parenchymal [15]. Emphysema is also associated with higher risks of lung cancer development and poor prognosis [16]. Although ILAs and emphysema are risk factors for PPCs after various surgeries, including lung cancer resections [17–19], the relationships between ILAs, emphysema, PPCs, and esophagectomies have not been thoroughly examined. Only one previous study reported the prevalence of ILAs in esophageal cancer patients (7%) [20]. Thus, limited information is available regarding the prevalence and risk factors for PPCs in patients with ILA or emphysema and esophageal cancer. We hypothesize that the extent of ILAs and emphysema influence the prevalence of PPCs after esophagectomy, even when combining other established risk factors. This study aimed to determine the effects of quantified diffuse pulmonary disease, including ILA and emphysema, on PPCs in esophageal cancer patients undergoing esophagectomy. Deep learning-based automated software was employed to objectively quantify diffuse pulmonary disease on chest CT images.

## Materials and methods

### Study population

**Fig. 1:**
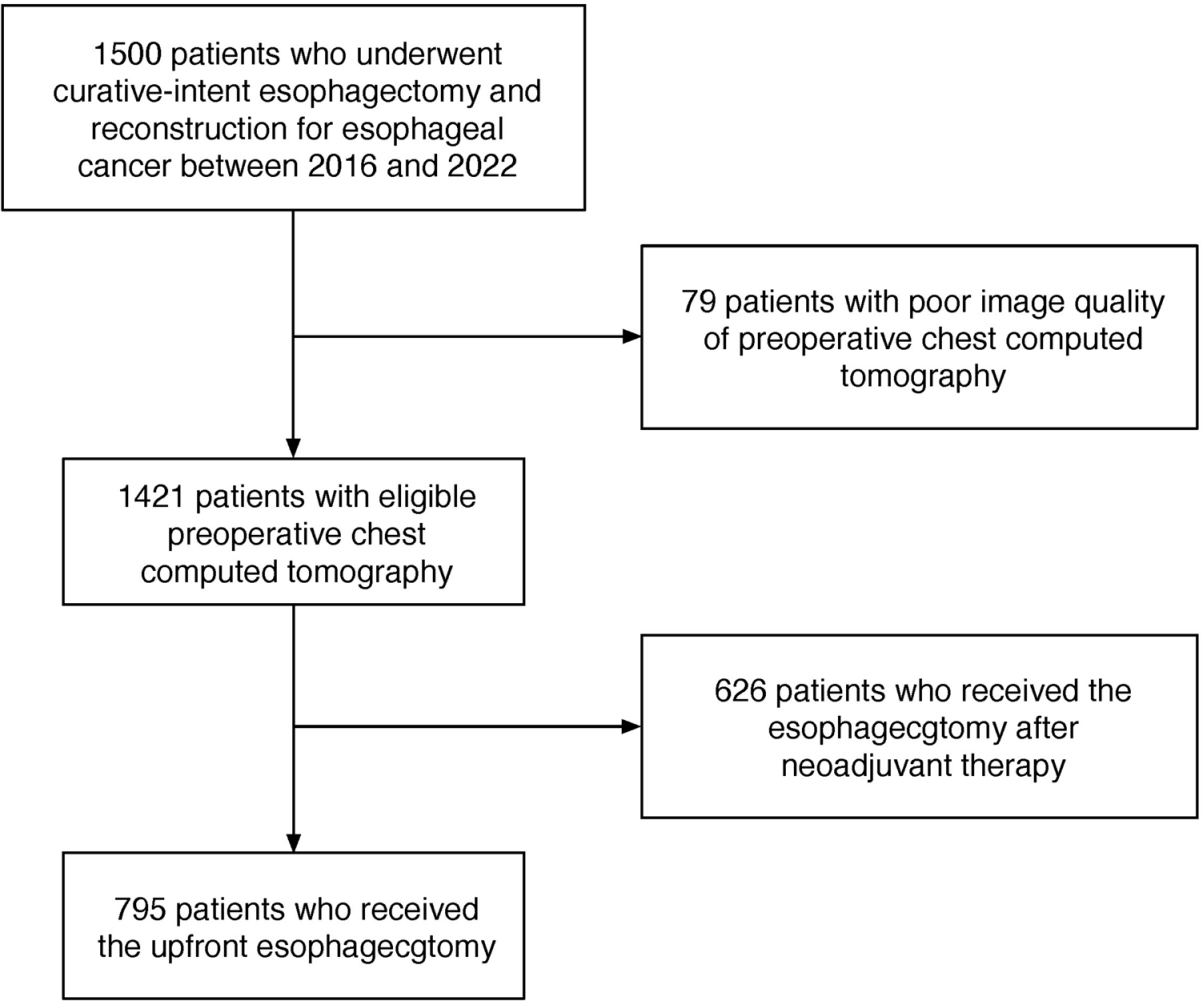
Flowchart of patient selection

Patients who underwent curative-intent esophagectomy and reconstruction for esophageal cancer between 2016 and 2022 were selected from the Registry for Thoracic Cancer Surgery at Samsung Medical Center (*n* = 1500). The exclusion criteria were as follows: 1) patients with inadequate CT images for quantitative analysis (the absence of volumetric images or the presence of thin-section images with a slice thickness greater than 2 mm) and 2) patients received neoadjuvant therapy (neoadjuvant therapy is a risk factor for pulmonary complications after esophagectomy) [21]. After excluding patients, 765 patients who underwent upfront esophagectomy for esophageal cancer were included in the analysis (Fig. 1). This study was approved by the Institutional Review Board of Samsung Medical Center (IRB no.: 2024-01-128) and was exempted from the requirement for informed consent because only de-identified data retrieved from electronic medical records were used in the study.

### Preoperative evaluation

Clinical characteristics, including age at surgery, sex, smoking status, pulmonary function test, tumor pathology and location, pathologic stage, and type of surgery, were obtained from the electronic medical records. The routine preoperative evaluation included a pulmonary function test, a chest CT scan, a positron emission tomography/CT scan, esophagogastroduodenoscopy, and endoscopic ultrasound. The pathological stage of esophageal cancer was defined based on the eighth edition of the TNM International Staging System [22]. Spirometry and DLCo measurements were performed using Vmax 22 spirometers (SensorMedics, Yorba Linda, CA, USA). Preserved pulmonary function was defined as a ratio of pre-bronchodilator FEV1 to forced vital capacity (FVC) > 0.70 and FVC ≥ 80% of the predicted value.

### CT acquisition and quantification

**Fig. 2:**
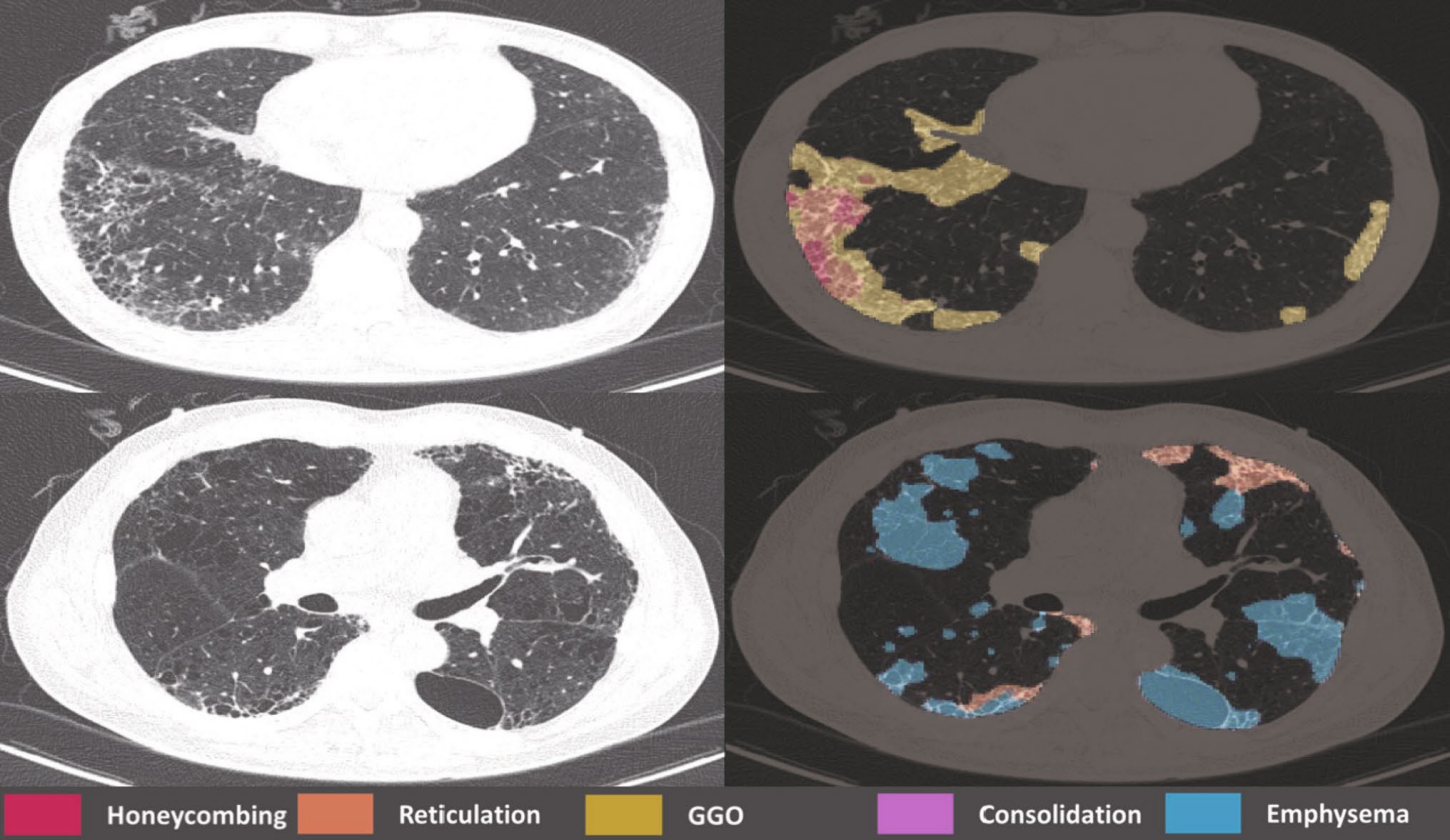
A representative example of texture analysis using the deep learning-based program

Preoperative CT scans were performed on two different 64-channel multi-slice CT scanners (General Electric, Chicago, IL, USA, and Siemens Healthcare, Munich, Germany). With the patient in the supine position, images were obtained at a voltage of 120 kVp and a slice thickness of 1–2 mmThe whole lung volume was automatically extracted to specifically delineate the parenchymal lung area, excluding pulmonary vessels and airways. The extent of ILA at the whole lung level was classified and quantified automatically using a deep learning-based approach. Using this approach, texture patterns were identified based on ground-glass opacity (GGO), reticulation, and honeycombing; the summation of the identified areas was divided by the total lung volume. Given that chest CT scans with focal or unilateral abnormalities affecting less than 5% of the lung area are considered indeterminate for ILA, we included these cases in our analysis as well [23–25]. The percentage of emphysema on CT was defined as the low attenuation area (density <  − 950 Hounsfield Units) divided by the total lung volume. Lung texture patterns were analyzed using CT images acquired with a sharp kernel. The percentage of emphysema on CT was defined as the low attenuation area (LAA, density <  − 950 Hounsfield Units) divided by the total lung volume-that is, LAA_-950_. When the target kernel images (sharp or soft) were unavailable, we utilized a DL-based kernel conversion method to obtain data that minimized quantification errors due to kernel differences [26–28]. The extracted texture patterns were precisely reviewed by an expert radiologist (H.Y.L., 20 years of experience). The entire quantitative process of kernel conversion, lung segmentation, and texture analysis was performed using commercial software based on a deep-learning algorithm (Aview, Coreline Soft, Seoul, South Korea) [29]. The quantitative analysis of a representative case is illustrated in Fig. 2.

### Surgical treatment of esophageal cancer

Esophagectomy with gastric reconstruction was performed using routine surgical procedures for esophageal cancer. All patients included in this study underwent right transthoracic esophagectomy under left single-lung ventilation. Open thoracotomies or minimally invasive esophagectomies were performed according to the surgeon’s preference. An intrathoracic anastomosis was preferred, but cervical anastomosis was performed if the resection margin was not sufficient. Transmediastinal, transhiatal, or transcervical approaches that allow bilateral lung ventilation were not included in this cohort.

### Postoperative pulmonary complications

PPCs included postoperative pneumonia, ALI, and ARDS that developed in the hospital during the postoperative period [30]. Although aspiration pneumonia may overlap clinically with postoperative pneumonia, aspiration and aspiration-related pneumonia were not classified as PPCs in this study. Given the clinical difficulty in clearly distinguishing aspiration pneumonia from postoperative infectious pneumonia after esophagectomy, we applied an operational definition primarily based on timing. Pneumonia developing after the initiation of oral intake was considered aspiration-related and excluded from PPCs when accompanied by clinical features suggestive of aspiration, reflecting the typical timing of aspiration events after oral feeding is resumed. Pneumonia was diagnosed if the patient received antibiotics for a suspected respiratory infection with new pulmonary infiltration detected on chest plain radiography and at least one of the following criteria occurred: new onset or changed sputum, fever, leukocytosis (> 12,000/μL), or documented pathogen in the sputum culture. ALI/ARDS was diagnosed when the patient met at least one of the following criteria: PaO_2_ < 60 mm Hg on room air, PaO_2_ to inspired oxygen fraction < 300, or arterial oxyhemoglobin saturation < 90% measured with pulse oximetry.

### Statistical analysis

Normally distributed continuous variables are presented as means ± standard deviations and compared using two-sample Student’s *t*-tests. Skewed variables are presented as medians with interquartile ranges and compared using Mann–Whitney *U* tests. Categorical variables were compared using chi-square and Fisher’s exact tests. The relationships between pulmonary function and parameters measured by texture analysis were determined using the Spearman correlation coefficient. Univariable logistic regression analyses were first performed to identify potential risk factors for PPC. Variables with a P value < 0.10 in the univariable analysis and clinically relevant variables were entered into the multivariable logistic regression model. Variable selection was performed using backward stepwise selection based on the Akaike Information Criterion (AIC), which balances model fit and complexity by minimizing − 2 log-likelihood plus a penalty term proportional to the number of parameters (k = 2). Age was modeled using a piecewise logistic regression approach with a cutoff at 65 years, allowing different linear age effects before and after this threshold. The extent of ILA (%) and extent of emphysema (%) showed skewed distribution, so we applied the logarithmic transformation to the normal distribution for logistic regression. A *p* value of < 0.05 was considered statistically significant. All statistical tests were performed using R version 4.3.1 (R Foundation for Statistical Computing, Vienna, Austria; www.r-project.org) and STATA 16 (StataCorp LLC, College Station, TX).

## Results

### Basic characteristics of patients

Patient demographics and baseline characteristics are shown in Table 1. The mean age of the patients was 64.71 ± 8.26 years, 91.2% were males, and 84.5% were former or current smokers. Squamous cell carcinoma was the most common tumor histology (*n* = 723, 94.5%), followed by adenocarcinoma (*n* = 35, 4.5%). Two-thirds (503, 65.7%) of patients underwent esophagectomy via open thoracotomy.

**Table 1:** Basic characteristics of patients (*n* = 765)

	No PPC (*n* = 636)	PPC (*n* = 129)	*p*-value
Age	64.48 ± 8.09	65.84 ± 9.02	0.089
Male	576 (90.6%)	122 (94.6%)	0.142
Smoking status			< 0.001
Never smoker	81 (12.7%)	6 (4.7%)	
Ex-smoker	300 (47.2%)	43 (33.3%)	
Current smoker	255 (40.1%)	80 (62.0%)	
FEV_1_%	90.69 ± 14.28	85.74 ± 16.40	0.0005
DLco %	87.25 ± 17.91	78.04 ± 15.14	< 0.001
FEV_1_/FVC	73.53 ± 8.54	69.70 ± 10.25	< 0.001
Pathology			0.325
Squamous cell carcinoma	598 (94.03%)	125 (96.9%)	
Adenocarcinoma	31 (4.87%)	4 (3.10%)	
Others	7 (1.10%)	0	
Location of lesion			0.476
Cervical	5 (0.79%)	0	
Upper	81 (12.74%)	15 (11.63%)	
Mid	271 (42.61%)	50 (38.76%)	
Lower	244 (38.36%)	59 (45.73%)	
EG junction	35 (5.5%)	5 (3.88%)	
pT			0.167
Tis	7 (1.1%)	0	
T1a	92 (14.5%)	14 (10.9%)	
T1b	348 (54.7%)	67 (51.9%)	
T2	84 (13.2%)	27 (20.9%)	
T3	100 (15.8%)	21 (16.3%)	
T4a	4 (0.7%)	0	
pN			0.164
N0	399 (62.8%)	82 (63.6%)	
N1	162 (25.6%)	25 (19.4%)	
N2	54 (8.5%)	19 (14.7%)	
N3	18 (2.8%)	3 (2.3%)	
Nx	2 (0.3%)	0	
Minimally invasive esophagectomy	237 (37.3%)	25 (19.4%)	< 0.001
Level of anastomosis			< 0.001
Intrathoracic anastomosis	380 (59.8%)	100 (77.5%)	
Cervical anastomosis	256 (40.3%)	29 (22.5%)	
Operation time (minutes)	265.04 ± 67.26	269.48 ± 67.71	0.494
Estimated blood loss (mL)	162.73 ± 155.55	149.68 ± 114.24	0.366
In-hospital mortality	2 (0.3%)	1 (0.8%)	0.445

Patients with PPCs were more likely to be current smokers and exhibited lower FEV_1_ (%) and DLco (%) compared to patients without PPCs. Additionally, patients with PPCs were more likely to have undergone open esophagectomy and intrathoracic anastomosis. Operative time and estimated blood loss did not differ significantly between patients with and without PPCs.

### Development of PPC

Among the 765 patients included in the study, 129 (16.9%) developed PPCs. Pneumonia, ALI, and ARDS occurred in 112 (14.6%), 29 (3.8%), and 8 (1.0%) patients, respectively, and 20 patients (14 with ALI and 6 with ARDS) suffered from both pneumonia and lung injury.

### Parameters of lung texture by automated software

The parameters of lung texture measured by the automated software are shown in Table 2. The whole lung volumes were not different between patients with and without PPCs. The extents of ILA (0.149 [0.062–0.502] vs. 0.342 [0.126–0.821], *p* < 0.001) and emphysema (0.123 [0.036–0.462] vs. 0.236 [0.033–1.367], *p* = 0.005) were significantly higher in patients with PPCs compared with patients without PPCs. To further investigate the differential impact of fibrotic and non-fibrotic ILAs, we separately analyzed the extent GGO, reticulation, and honeycombing in the PPC and no PPC groups (Table 2). Patients with PPC exhibited a significantly higher median extent of GGO compared to those without PPC (*p* < 0.001). Similarly, the extent of reticulation, a fibrotic ILA feature, was higher in the PPC group than in the no PPC group (*p* < 0.001). Honeycombing, which represents a more advanced fibrotic feature, was also significantly more prevalent in the PPC group compared to the no PPC group (*p* < 0.001).

**Table 2:** Parameters measured by the automated analysis of computed tomography scans

	No PPC (*n* = 636)	PPC (*n* = 129)	p*
Whole lung volume (mL)	4920.561 (4247.186–5629.445)	4794.276 (4106.011–5608.299)	0.804
Right lung volume (mL)	2644.135 (2300.51–3033.494)	2652.793 (2272.179–3083.135)	0.955
Right upper lobe volume (mL)	1013.81 (850.737–1169.711)	990.387 (839.395–1136.236)	0.304
Right middle lobe volume (mL)	429.434 (333.994–530.272)	419.067 (326.337–512.908)	0.527
Right lower lobe volume (ml)	1200.886 (977.629–1456.623)	1243.339 (982.409–1482.713)	0.385
Left lung volume (mL)	2233.531 (1916.468–2581.594)	2223.958 (1818.833–2625.76)	0.771
Left upper lobe volume (mL)	1205.076 (1046.944–1382.640)	1195.097 (1036.973–1380.415)	0.837
Left lower lobe volume (mL)	1028.455 (823.698–1243.459)	1028.86 (772.313–1248.134)	0.773
Ground glass opacity (%), unit: × 10^−3^	98.837 (41.056–289.550)	211.111 (69.399–588.326)	< 0.001
Reticulation (%), unit: × 10^−3^	39.094 (11.932–131.261)	96.273 (29.302–272.917)	< 0.001
Honeycombing (%), unit: × 10^−3^	0.098 (0–0.533)	0.313 (0.028–1.333)	< 0.001
Extent of ILA (%)	0.149 (0.062–0.502)	0.342 (0.126–0.821)	< 0.001
Extent of emphysema (%) of whole lung	0.123 (0.036–0.462)	0.236 (0.033–1.367)	0.005
Extent of emphysema (%) of right lung	0.110 (0.030–0.400)	0.199 (0.026–0.972)	0.018
Extent of emphysema (%) of left lung	0.129 (0.034–0.459)	0.238 (0.390–0.158)	0.003

All parameters are presented as median (interquartile range)

* Mann–Whitney U test

**Fig. 3:**
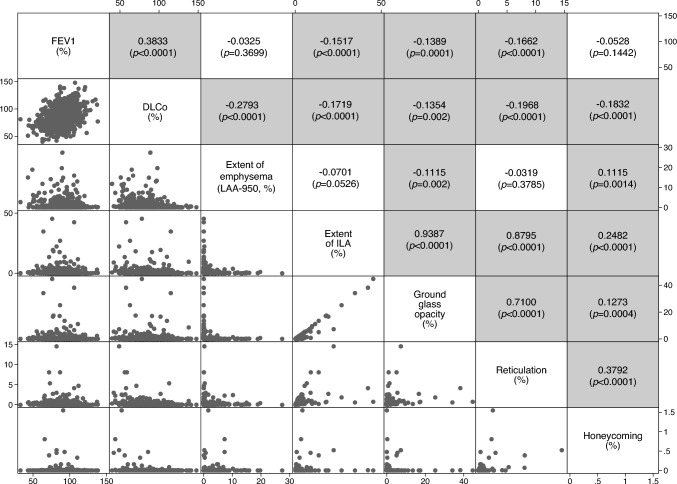
Pairwise correlations between pulmonary function parameters and quantitative CT-derived imaging metrics. The lower-left panels show scatter plots of individual patient data, with each dot representing one patient. The upper-right panels display correlation coefficients (Spearman’s ρ) with corresponding p-values. Gray-shaded boxes indicate statistically significant correlations (p < 0.05). Variables shown include FEV1 (% predicted), DLCO (% predicted), low-attenuated area (LAA-950, %), extent of interstitial lung abnormality (ILA, %), ground-glass opacity (%), reticulation (%), and honeycombing (%). Each scatter plot displays the relationship between the two variables indicated by its row and column labels along the diagonal

The correlation of texture parameters with lung functions is illustrated in Fig. 3. FEV_1_ (%) showed a significant correlation with the extent of ILA, but not with the extent of emphysema. The extent of ILA correlated with DLco (%) and other texture parameters. In addition, the correlations between whole lung volume, extent of ILA and emphysema were documented in Supplementary Fig. S1. The whole lung volume showed the positive correlation with extent of emphysema (Spearman correlation coefficient 0.2340, *p* < 0.05), but negative correlation with extent of ILA (Spearman correlation coefficient −0.3871, *p* < 0.05).

### Factors associated with the development of PPCs

**Fig. 4:**
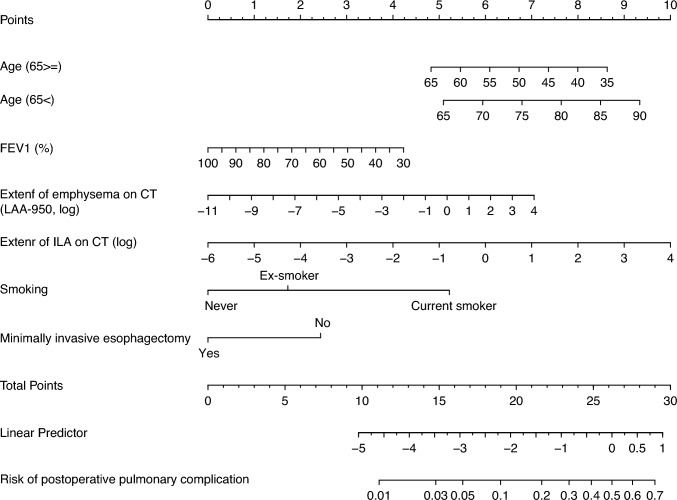
Nomogram predicting the risk of pulmonary complication

The univariable and multivariable analyses to identify factors associated with PPCs are shown in Table 3. The extent of ILA and emphysema were not normally distributed. Therefore, the data were logarithmically transformed to achieve normal distribution before the univariable analyses. Univariable analyses revealed that smoking status (current smoker), type of approach (open thoracotomy), FEV_1_ (%), DLco (%), and the extent of ILA and emphysema were associated with the development of PPC (Table 3). Age was not related to PPC (*p* = 0.090), but age > 65 years was a significant risk factor for PPC in the univariable analysis. As a correlation was detected between FEV1 (%) and DLco (%), only FEV_1_ (%) was included in the multivariable analysis. In patients > 65 years, age tended to be associated with the development of PPCs (odds ratio [OR] 1.054, *p* = 0.062). The multivariable logistic analysis demonstrated that preoperative emphysema (OR 1.158, *p* = 0.004) and ILA (OR 1.364, *p* < 0.001) were risk factors for PPC after adjusting for other confounding factors. The nomogram which can predict the risk of PPC was constructed based on the multivariable analysis (Fig. 4).

**Table 3:** Univariable and multivariable analyses of postoperative pulmonary complications

	Univariable analysis		Multivariable analysis	
	Odds ratio (95% confidence interval)	*p*	Odds ratio (95% confidence interval)	*p*
Sex	0.551 (0.246–1.234)	0.147	…	…
Age	1.020 (0.997–1.044)	0.090	…	…
Age (< 65, 1 year increase)	0.988 (0.935 ~ –1.045)	0.674	0.967 (0.912–1.025)	0.258
Age ($\ge$ 65, 1 year increase)	1.044 (0.993–1.097)	0.0906	1.054 (0.997–1.114)	0.062
Smoking status				
Ex-smoker (vs. non-smoker)	1.935 (0.795–4.705)	0.145	1.680 (0.667–4.236)	0.271
Current smoker (vs. non-smoker)	4.235 (1.780–10.073)	0.001	4.887 (1.962–12.169)	0.001
Minimally invasive esophagectomy (vs. open esophagectomy)	0.430 (0.276–0.670)	< 0.001	0.485 (0.304–0.775)	0.003
FEV_1_ (%)	0.977 (0.965–0.990)	0.001	0.981 (0.966–0.997)	0.019
Extent of ILA (%, log scale)	1.049(1.002–1.097)	0.039	1.364 (1.182–1.573)	< 0.001
Extent of emphysema (%, log scale)	1.114 (1.045–1.189)	0.001	1.158 (1.049–1.279)	0.004

## Discussion

The influence of ILA and emphysema, identified using advanced deep-learning algorithm-based software, on the occurrence of PPCs was assessed in patients with esophageal cancer undergoing esophagectomies. Multivariable logistic regression demonstrated a significant association between the extent of ILA and emphysema and PPCs, even after accounting for potential confounding factors. To the best of our knowledge, this is the first study to analyze the impact of ILA and emphysema on treatment-related complications in patients with esophageal cancer who underwent curative surgical treatment, using automated deep learning-enhanced software.

ILAs are characterized by incidental findings on chest CT scans and defined as interstitial abnormalities in more than 5% of any lung zone, without prior suspicion of ILD [10]. Systematic evaluations of large cohorts demonstrated that ILAs are linked to poor clinical outcomes, including increased respiratory symptoms and higher overall mortality [13, 25, 31, 32]. Furthermore, even in the absence of clinical interstitial lung disease or fibrosis using a 5% threshold, subtle visually and objectively defined interstitial features are frequently shown in smokers with emphysema and turned out to have worse clinical disease severity and mortality than those with emphysema alone. Meanwhile, numerous studies demonstrated that ILAs are a risk factor for PPCs and mortality in patients with early-stage lung cancer, and ILAs are associated with high mortality in patients with advanced lung cancer [14]. The frequency of PPCs in patients with esophageal cancer and lung cancer are similar. Thus, understanding the impact of ILAs on surgical outcomes in patients with esophageal cancer is imperative. However, studies examining the influence of ILA on treatment outcomes in esophageal cancer are scarce. Tseng et al. reported a 7% prevalence of ILAs among 208 esophageal cancer patients, consistent with the rates observed in the general and smoking populations [20]. However, this study encompassed patients with locally advanced esophageal cancer, and most patients were undergoing concurrent chemoradiotherapy, which may induce or exacerbate ILA or ILD and prevent the accurate assessment of the impact of ILA on PPCs. Our results indicate that ILA, including indeterminate for ILA elevates the risk of PPCs in patients with esophageal cancer undergoing surgical treatment.

ILAs may represent subclinical stages of various interstitial lung diseases or early manifestations of conditions such as idiopathic pulmonary fibrosis or interstitial pneumonia [33]. These diseases may progress following esophagectomy, reminiscent of the postoperative acute exacerbations in lung cancer patients undergoing surgical resection [34]. Although the pathogenesis of postoperative ALI and acute exacerbation of IPF are unclear, excessive inflammation, triggered by surgery, medications, or aspiration, may lead to alveolar epithelial tissue damage [35–37]. The detrimental impact of ILA on the outcomes of surgical interventions for esophageal cancer highlights the importance of additional studies aimed at establishing optimal surveillance and treatment strategies for esophageal cancer patients with ILA.

The clinical concern regarding ILA on chest CT was initially proposed based on visual assessment in the reference below, and it has been shown to remain useful to this day. However, the Fleischner group’s initial visual assessment method has several prerequisites, including an evaluation based on only three images taken at specific lung levels [10]. This approach was attempted because a volumetric, consistent assessment of the whole lung cannot be achieved visually. Chae et al. reported that the inter-reader agreement on ILA imaging features was 0.47–0.81 [38]. To address this challenge, various methods have been explored to quantify the extent of ILA, including density measurements of highly attenuated regions, local histogram analysis, and deep learning-based texture evaluation [13]. The strength of our study lies in the utilization of automated deep learning-based tissue analysis to quantify the extent of ILA. In other words, we opted to use automated quantification methods using software for our study. The primary reason for this choice was the importance of quickly obtaining relevant and reproducible quantitative values, which can be applied to patients in clinical fields, rather than addressing the disagreements in ILA severity assessments among chest radiologists from different hospitals with varying levels of ILA experience. The objectivity of this method has been validated [29]. Consequently, employing this advanced approach enabled us to predict PPC risk following esophagectomy using quantified data rather than subjective assessments. Our findings revealed that even indeterminate ILA, comprising less than 5% of the lung, was significantly associated with PPC, underscoring the substantial impact of even indeterminate for ILA on postoperative outcomes in patients with esophageal cancer. Recent study also identified a 1.8%-3.6% extent threshold per lung zone using the deep learning-based texture analysis for ILA evaluation and showed better sensitivity and specificity for visually detected ILA by thoracic radiologists.[39, 40] Thus, incorporating identification of the presence of interstitial features as well as quantification of ILA into preoperative evaluations to identify patients at heightened risk of PPCs enables clinicians to improve surgical outcomes.

Emphysema is characterized by irreversible lung tissue destruction and airspace enlargement and can be detected on chest CT. Detection of emphysema, particularly in individuals with normal spirometry, predicted the risk of PPCs in this study. Thus, the detection of emphysema is crucial for clinical decision-making and provides insights into lung structural abnormalities that may not be apparent through conventional pulmonary function assessments [41]. Automated density methods enhance the reliability and sensitivity of emphysema quantification [42], facilitating accurate risk stratification and personalized management strategies for individuals undergoing surgical procedures. Therefore, integrating CT-based assessment of emphysema into preoperative evaluations will enable clinicians to identify high-risk individuals, optimize perioperative care, and improve patient outcomes by mitigating the risk of PPCs.

In addition to patient-related pulmonary risk factors, surgical approach and operative techniques may also influence the occurrence of postoperative pulmonary complications. In our cohort, intrathoracic anastomosis was associated with a higher incidence of PPCs; however, this finding should be interpreted with caution. During a substantial portion of the study period, intrathoracic anastomosis at our institution was predominantly performed as part of an open Ivor–Lewis esophagectomy under thoracotomy. Consequently, most patients who underwent intrathoracic anastomosis were exposed to open thoracic surgery, which is known to increase pulmonary morbidity. Therefore, the observed association between intrathoracic anastomosis and PPCs in this study likely reflects the confounding effect of surgical approach rather than an intrinsic risk related to the anastomotic location itself.

This study had several limitations. The retrospective, single-center design in an Asian population limits the generalizability of our findings. Additionally, the exclusive focus on patients from tertiary hospitals may limit the extrapolation of our results to broader healthcare contexts. Despite these limitations inherent to single-institution studies, the inclusion of a relatively large cohort of patients with esophageal cancer contributes to the robustness and clinical applicability of our findings. Further exploration and validation in diverse healthcare settings are warranted to verify the broader implications of our findings.

In addition, patients who underwent neoadjuvant chemotherapy were intentionally excluded from this analysis. Although neoadjuvant therapy followed by surgery is currently the standard treatment strategy for advanced esophageal cancer, preoperative CT scans obtained after neoadjuvant treatment often demonstrate heterogeneous timing, variable image quality, and treatment-related lung parenchymal changes. These factors may confound automated CT-based quantification of ILA and emphysema. Therefore, our findings are primarily applicable to patients undergoing upfront surgery, and caution is required when extrapolating these results to populations treated with neoadjuvant therapy. Future studies incorporating larger cohorts of patients receiving neoadjuvant treatment are warranted to clarify the interaction between treatment-related lung changes and PPCs.

To elaborate further, even though our study primarily aimed to confirm the hypothesis that the severity of ILA and LAA is an important predisposing factor for pulmonary complications immediately following esophageal cancer surgery, if we could propose specific thresholds that can be used in practice, it would be very beneficial. In order to propose usable thresholds, particularly validation through external cohorts must be presented. While we classified minimal honeycombing as part of the ILA spectrum in our analysis, we acknowledge that the presence of honeycombing may represent early stages of ILD. This is because microscopic honeycombing may exist at the histopathologic level; however, in terms of CT dimensions, there is still a degree of ILA where honeycombing is not visible. Consequently, as highlighted in prior literature, the distinction between ILA, early ILD, and mild ILD can be artificial, and these categories may reflect different stages of the same disease continuum [43]. The progression of ILA findings into clinically significant ILD remains an area of ongoing research, and further studies are needed to better characterize the clinical implications and natural history of ILA, especially in surgical populations.

In conclusion, our study demonstrates that the extent of ILAs, objectively quantified through automated texture analysis using a deep-learning algorithm, and emphysema detected by CT are significantly associated with the occurrence of PPCs after esophagectomy in patients with esophageal cancer. Our results highlight the utility of preoperative CT for assessing postoperative risk. Furthermore, our findings suggest that evaluating the extent of ILA and emphysema is important for surgical planning and improving the outcomes of esophagectomy.

## Supplementary Information

Below is the link to the electronic supplementary material.

Supplementary Fig. S1. Pairwise correlations among whole lung volume and quantitative CT-derived imaging metrics. The lower-left panels show scatter plots of individual patient data, with each dot representing one patient. The upper-right panels display correlation coefficients (Spearman’s ρ) with corresponding p-values. Variables include whole lung volume (mL), extent of interstitial lung abnormality (ILA, %), and low-attenuated area (%). Axes represent the corresponding variables indicated on the diagonal. Statistically significant correlations (p < 0.05) are indicated with p-values in the upper panels (PDF 345 KB)

## Data Availability

The datasets used and/or analyzed during the current study are available from the corresponding author upon reasonable request.

## Abbreviations

ALI: Acute lung injury
ARDS: Acute respiratory distress syndrome
CT: Computed tomography
DL_CO_: Diffusing capacity for carbon monoxide
FEV_1_: Forced expiratory volume in one second
FVC: Forced vital capacity
GGO: Ground-glass opacity
ILAs: Interstitial lung abnormalities
ILD: Interstitial lung disease
LAA_-950_: Low attenuating area below -950HU on CT
PPCs: Postoperative pulmonary complications

## References

[1] Takeuchi H, Miyata H, Gotoh M, et al. A risk model for esophagectomy using data of 5354 patients included in a Japanese nationwide web-based database. Ann Surg. 2014;260(2):259–66. 24743609 10.1097/SLA.0000000000000644

[2] Yoshida N, Yamamoto H, Baba H, et al. Can minimally invasive esophagectomy replace open esophagectomy for esophageal cancer? Latest analysis of 24,233 esophagectomies from the Japanese National Clinical Database. Ann Surg. 2020;272(1):118–24. 30720501 10.1097/SLA.0000000000003222

[3] Yang CK, Teng A, Lee DY, et al. Pulmonary complications after major abdominal surgery: National Surgical Quality Improvement Program analysis. J Surg Res. 2015;198(2):441–9. 25930169 10.1016/j.jss.2015.03.028

[4] Yoshida N, Harada K, Iwatsuki M, et al. Precautions for avoiding pulmonary morbidity after esophagectomy. Ann Gastroenterol Surg. 2020;4(5):480–4. 33005841 10.1002/ags3.12354 PMC7511556

[5] Markar S, Gronnier C, Duhamel A, et al. Pattern of postoperative mortality after esophageal cancer resection according to center volume: results from a Large European Multicenter Study. Ann Surg Oncol. 2015;22(8):2615–23. 25605511 10.1245/s10434-014-4310-5

[6] Baba Y, Yoshida N, Shigaki H, et al. Prognostic impact of postoperative complications in 502 patients with surgically resected esophageal squamous cell carcinoma: a retrospective single-institution study. Ann Surg. 2016;264(2):305–11. 26670288 10.1097/SLA.0000000000001510

[7] Saeki H, Tsutsumi S, Tajiri H, et al. Prognostic significance of postoperative complications after curative resection for patients with esophageal squamous cell carcinoma. Ann Surg. 2017;265(3):527–33. 28169928 10.1097/SLA.0000000000001692

[8] Manara M, Bona D, Bonavina L, et al. Impact of pulmonary complications following esophagectomy on long-term survival: multivariate meta-analysis and restricted mean survival time assessment. Updates Surg. 2024;76(3):757–67. 38319522 10.1007/s13304-024-01761-2 PMC11129973

[9] Goense L, Meziani J, Bulbul M, et al. Pulmonary diffusion capacity predicts major complications after esophagectomy for patients with esophageal cancer. Dis Esophagus. 2019;32(3):doy082. 30239639 10.1093/dote/doy082

[10] Hatabu H, Hunninghake GM, Richeldi L, et al. Interstitial lung abnormalities detected incidentally on CT: a position paper from the Fleischner Society. Lancet Respir Med. 2020;8(7):726–37. 32649920 10.1016/S2213-2600(20)30168-5 PMC7970441

[11] Tsushima K, Sone S, Yoshikawa S, et al. The radiological patterns of interstitial change at an early phase: over a 4-year follow-up. Respir Med. 2010;104(11):1712–21. 20538446 10.1016/j.rmed.2010.05.014

[12] Araki T, Putman RK, Hatabu H, et al. Development and progression of interstitial lung abnormalities in the Framingham Heart Study. Am J Respir Crit Care Med. 2016;194(12):1514–22. 27314401 10.1164/rccm.201512-2523OC PMC5215030

[13] Hata A, Schiebler ML, Lynch DA, et al. Interstitial lung abnormalities: state of the art. Radiology. 2021;301(1):19–34. 34374589 10.1148/radiol.2021204367 PMC8487219

[14] Araki T, Dahlberg SE, Hida T, et al. Interstitial lung abnormality in stage IV non-small cell lung cancer: a validation study for the association with poor clinical outcome. Eur J Radiol Open. 2019;6:128–31. 30984804 10.1016/j.ejro.2019.03.003 PMC6444119

[15] Snider GL, Kleinerman J, Thurlbeck WM, et al. The definition of emphysema. Report of a National Heart, Lung, and Blood Institute, Division of Lung Diseases workshop. Am Rev Respir Dis. 1985;132(1):182–5. 4014865 10.1164/arrd.1985.132.1.182

[16] Yang X, Wisselink HJ, Vliegenthart R, et al. Association between chest CT-defined emphysema and lung cancer: a systematic review and meta-analysis. Radiology. 2022;304(2):322–30. 35503012 10.1148/radiol.212904

[17] Jeong WG, Kim YH, Lee JE, et al. Predictive Value of Interstitial Lung Abnormalities for Postoperative Pulmonary Complications in Elderly Patients with Early-stage Lung Cancer. Cancer Res Treat. 2022;54(3):744–52. 34583454 10.4143/crt.2021.772 PMC9296932

[18] Im Y, Park HY, Shin S, et al. Prevalence of and risk factors for pulmonary complications after curative resection in otherwise healthy elderly patients with early stage lung cancer. Respir Res. 2019;20(1):136. 31272446 10.1186/s12931-019-1087-x PMC6610954

[19] Shin S, Park HY, Kim H, et al. Joint effect of airflow limitation and emphysema on postoperative outcomes in early-stage nonsmall cell lung cancer. Eur Respir J. 2016;48(6):1743–50. 27811074 10.1183/13993003.01148-2016

[20] Tseng SC, Hino T, Hatabu H, et al. Interstitial lung abnormalities in patients with locally advanced esophageal cancer: prevalence, risk factors, and clinical implications. J Comput Assist Tomogr. 2022;46(6):871–7. 35995596 10.1097/RCT.0000000000001366 PMC9675694

[21] Chen X, Du M, Tang H, et al. Comparison of pulmonary function changes between patients receiving neoadjuvant chemotherapy and chemoradiotherapy prior to minimally invasive esophagectomy: a randomized and controlled trial. Langenbecks Arch Surg. 2022;407(7):2673–80. 36006505 10.1007/s00423-022-02646-x PMC9640419

[22] Rice TW, Ishwaran H, Ferguson MK, et al. Cancer of the esophagus and esophagogastric junction: an eighth edition staging primer. J Thorac Oncol. 2017;12(1):36–42. 27810391 10.1016/j.jtho.2016.10.016 PMC5591443

[23] Washko GR, Lynch DA, Matsuoka S, et al. Identification of early interstitial lung disease in smokers from the COPDGene Study. Acad Radiol. 2010;17(1):48–53. 19781963 10.1016/j.acra.2009.07.016 PMC2790552

[24] Hunninghake GM, Hatabu H, Okajima Y, et al. MUC5B promoter polymorphism and interstitial lung abnormalities. N Engl J Med. 2013;368(23):2192–200. 23692170 10.1056/NEJMoa1216076 PMC3747636

[25] Putman RK, Gudmundsson G, Axelsson GT, et al. Imaging patterns are associated with interstitial lung abnormality progression and mortality. Am J Respir Crit Care Med. 2019;200(2):175–83. 30673508 10.1164/rccm.201809-1652OC PMC6635786

[26] Kim H, Oh G, Beom Seo J, et al. Multi-domain CT translation by a routable translation network. Phys Med Biol. 2022;67(21):215002. 10.1088/1361-6560/ac950e 36162401

[27] Hwang HJ, Kim H, Seo JB, et al. Generative adversarial network-based image conversion among different computed tomography protocols and vendors: effects on accuracy and variability in quantifying regional disease patterns of interstitial lung disease. Korean J Radiol. 2023;24(8):807–20. 37500581 10.3348/kjr.2023.0088 PMC10400368

[28] Lee SM, Lee JG, Lee G, et al. CT Image Conversion among Different Reconstruction Kernels without a Sinogram by Using a Convolutional Neural Network. Korean J Radiol. 2019;20(2):295–303. 30672169 10.3348/kjr.2018.0249 PMC6342751

[29] Kim MS, Choe J, Hwang HJ, et al. Interstitial lung abnormalities (ILA) on routine chest CT: comparison of radiologists’ visual evaluation and automated quantification. Eur J Radiol. 2022;157:110564. 36308851 10.1016/j.ejrad.2022.110564

[30] Miskovic A, Lumb AB. Postoperative pulmonary complications. Br J Anaesth. 2017;118(3):317–34. 28186222 10.1093/bja/aex002

[31] Axelsson GT, Putman RK, Aspelund T, et al. The associations of interstitial lung abnormalities with cancer diagnoses and mortality. Eur Respir J. 2020;56(6):1902154. 32646918 10.1183/13993003.02154-2019 PMC7876778

[32] Doyle TJ, Washko GR, Fernandez IE, et al. Interstitial lung abnormalities and reduced exercise capacity. Am J Respir Crit Care Med. 2012;185(7):756–62. 22268134 10.1164/rccm.201109-1618OC PMC3326424

[33] Miller ER, Putman RK, Vivero M, et al. Histopathology of interstitial lung abnormalities in the context of lung nodule resections. Am J Respir Crit Care Med. 2018;197(7):955–8. 28934558 10.1164/rccm.201708-1679LE PMC6020414

[34] Voltolini L, Bongiolatti S, Luzzi L, et al. Impact of interstitial lung disease on short-term and long-term survival of patients undergoing surgery for non-small-cell lung cancer: analysis of risk factors. Eur J Cardiothorac Surg. 2013;43(1):e17-23. 23129356 10.1093/ejcts/ezs560

[35] Jordan S, Mitchell JA, Quinlan GJ, et al. The pathogenesis of lung injury following pulmonary resection. Eur Respir J. 2000;15(4):790–9. 10780775 10.1034/j.1399-3003.2000.15d26.x

[36] Collard HR, Ryerson CJ, Corte TJ, et al. Acute exacerbation of idiopathic pulmonary fibrosis. An International Working Group Report. Am J Respir Crit Care Med. 2016;194(3):265–75. 27299520 10.1164/rccm.201604-0801CI

[37] Della Rocca G, Coccia C. Acute lung injury in thoracic surgery. Curr Opin Anaesthesiol. 2013;26(1):40–6. 23235524 10.1097/ACO.0b013e32835c4ea2

[38] Chae KJ, Chung MJ, Jin GY, et al. Radiologic-pathologic correlation of interstitial lung abnormalities and predictors for progression and survival. Eur Radiol. 2022;32(4):2713–23. 34984519 10.1007/s00330-021-08378-8

[39] Park S, Choe J, Hwang HJ, et al. Long-Term Follow-Up of Interstitial Lung Abnormality: Implication in Follow-Up Strategy and Risk Thresholds. Am J Respir Crit Care Med. 2023;208(8):858–67. 37590877 10.1164/rccm.202303-0410OC

[40] Chae KJ, Lim S, Seo JB, et al. Interstitial lung abnormalities at CT in the Korean national lung cancer screening program: prevalence and deep learning-based texture analysis. Radiology. 2023;307(4):e222828. 37097142 10.1148/radiol.222828

[41] Oh AS, Strand M, Pratte K, et al. Visual emphysema at chest CT in GOLD Stage 0 cigarette smokers predicts disease progression: results from the COPDGene study. Radiology. 2020;296(3):641–9. 32633676 10.1148/radiol.2020192429 PMC7457948

[42] Oh AS, Baraghoshi D, Lynch DA, et al. Emphysema progression at CT by deep learning predicts functional impairment and mortality: results from the COPDGene study. Radiology. 2022;304(3):672–9. 35579519 10.1148/radiol.213054 PMC9434819

[43] Tomassetti S, Poletti V, Ravaglia C, et al. Incidental discovery of interstitial lung disease: diagnostic approach, surveillance and perspectives. Eur Respir Rev. 2022;31(164):210206. 35418487 10.1183/16000617.0206-2021 PMC9488620

