# Supplementary figures and images for: Automated quantification of interstitial lung abnormalities and emphysema on computed tomography: a predictive marker for postoperative pulmonary complications after esophagectomy
Source: Esophagus. 2026 May 13;23(3):582–93. doi: 10.1007/s10388-026-01208-0 (PMC13319435; doi:10.1007/s10388-026-01208-0)

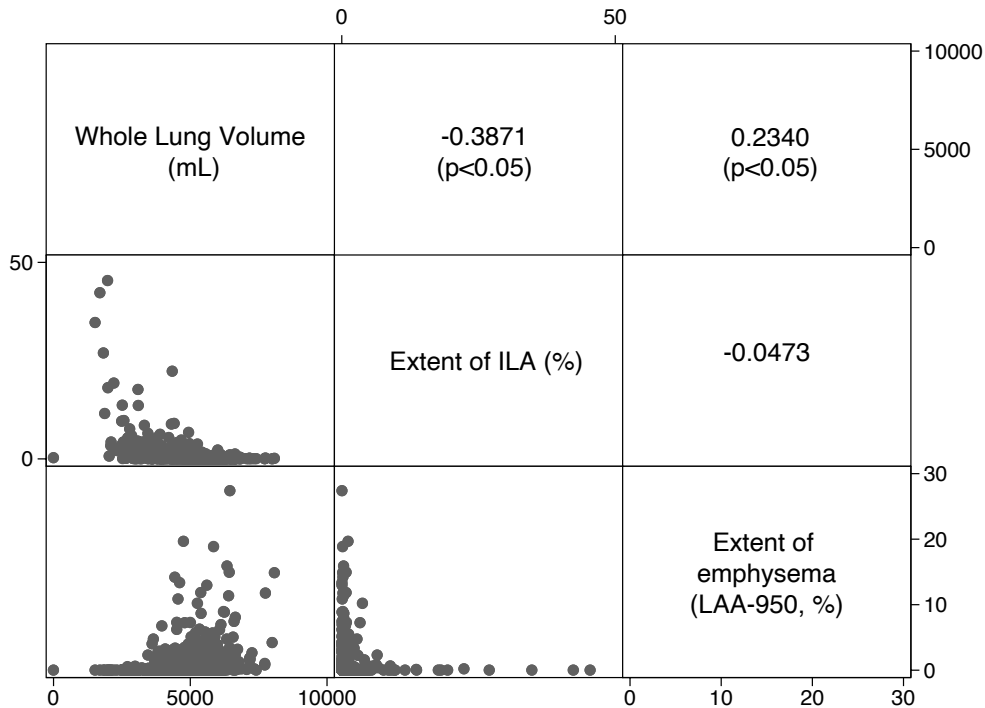

Supplement: Supplementary file 1 — Supplementary Fig. S1. Pairwise correlations among whole lung volume and quantitative CT-derived imaging metrics. The lower-left panels show scatter plots of individual patient data, with each dot representing one patient. The upper-right panels display correlation coefficients (Spearman’s ρ) with corresponding p-values. Variables include whole lung volume (mL), extent of interstitial lung abnormality (ILA, %), and low-attenuated area (%). Axes represent the corresponding variables indicated on the diagonal. Statistically significant correlations (p < 0.05) are indicated with p-values in the upper panels (PDF 345 KB) [file 10388_2026_1208_MOESM1_ESM.pdf]
